# High Working Memory Load Impairs Language Processing during a Simulated Piloting Task: An ERP and Pupillometry Study

**DOI:** 10.3389/fnhum.2016.00240

**Published:** 2016-05-25

**Authors:** Mickaël Causse, Vsevolod Peysakhovich, Eve F. Fabre

**Affiliations:** ^1^Département Conception et Conduite des Véhicules Aéronautiques et Spatiaux, Institut Supérieur de l’Aéronautique et de l’EspaceToulouse, France; ^2^Ecole de Psychologie, Université LavalQuébec, QC, Canada

**Keywords:** mental workload evaluation, electroencephalography/event-related potential (EEG/ERPs), pupil size, neuroergonomics, human factors, selective attention, attentional orienting

## Abstract

Given the important amount of visual and auditory linguistic information that pilots have to process, operating an aircraft generates a high working-memory load (WML). In this context, the ability to focus attention on relevant information and to remain responsive to concurrent stimuli might be altered. Consequently, understanding the effects of WML on the processing of both linguistic targets and distractors is of particular interest in the study of pilot performance. In the present work, participants performed a simplified piloting task in which they had to follow one of three colored aircraft, according to specific written instructions (i.e., the written word for the color corresponding to the color of one of the aircraft) and to ignore either congruent or incongruent concurrent auditory distractors (i.e., a spoken name of color). The WML was manipulated with an n-back sub-task. Participants were instructed to apply the current written instruction in the low WML condition, and the 2-back written instruction in the high WML condition. Electrophysiological results revealed a major effect of WML at behavioral (i.e., decline of piloting performance), electrophysiological, and autonomic levels (i.e., greater pupil diameter). Increased WML consumed resources that could not be allocated to the processing of the linguistic stimuli, as indexed by lower P300/P600 amplitudes. Also, significantly, lower P600 responses were measured in incongruent vs. congruent trials in the low WML condition, showing a higher difficulty reorienting attention toward the written instruction, but this effect was canceled in the high WML condition. This suppression of interference in the high load condition is in line with the engagement/distraction trade-off model. We propose that P300/P600 components could be reliable indicators of WML and that they allow an estimation of its impact on the processing of linguistic stimuli.

## Introduction

### Visual-Auditory Interference

Depending on the current task, the information surrounding us is roughly divided into relevant or irrelevant. Naturally, we tend to ignore the irrelevant information and to privilege that which is relevant. Despite such top-down attentional focus on a primary task, concurrent stimuli can capture human attention, especially when they share common characteristics with the focal task (Folk and Remington, 1998). As stated by Watkins et al. (2007), although distracting subjects, such attentional capture may be advantageous for survival because even a single stimulus can convey critical information about the environment. The distraction phenomenon has been widely investigated over the last decades (e.g., Parmentier, 2014). According to various authors, distraction may result from three different processing steps (Escera et al., 2000; Berti, 2008, 2013; Horváth et al., 2008). First, a pre-attentive change detection step may occur automatically when novel/deviant stimulus appears in the environment. Second, once the concurrent stimulus is detected, attentional resources may be automatically allocated to it (i.e., involuntary orienting of attention) at the expense of goal-relevant stimuli. Third, if the stimulus is irrelevant to the task, a voluntary reorientation of attentional resources from irrelevant stimulus to relevant stimulus may finally occur. These involuntary and voluntary shifts of attention are assumed to interfere with the processing of the information relevant to the task at hand. Generally, the literature on visual-auditory interference tends to support this three-step model using a wide variety of experimental paradigms such as auditory-visual oddball tasks (Andrés et al., 2006; Boll and Berti, 2009; Bendixen et al., 2010; Parmentier and Andrés, 2010; Ljungberg and Parmentier, 2012), task-irrelevant auditory distractor probes (Scheer et al., 2016), visual-auditory Stroop tasks (Roelofs, 2005; Donohue et al., 2013; Elliott et al., 2014), response competition paradigms (e.g., Lavie and Cox, 1997; Tellinghuisen and Nowak, 2003), and more ecological tasks like decision-making during aircraft landing (e.g., Scannella et al., 2013). Overall, auditory distractors are likely to interfere with the processing of visual targets as longer response times and sometimes a decrement in accuracy are observed (Stuart and Carrasco, 1993; Yuval-Greenberg and Deouell, 2009; Chen and Spence, 2011; Berti, 2013; Donohue et al., 2013).

The electroencephalography (EEG) technique is particularly appropriate for the study of the processing of auditory distractors in that it has a high temporal resolution which enables to observe the different steps of the process (Luck and Kappenman, 2011). The mismatch negativity (MMN) event related potential (ERP), a negative deflection occurring between 150 and 250 ms after stimulus onset, maximal at frontal and central sites, was found to be elicited by novel/deviant auditory distractors; this was interpreted as reflecting the pre-attentive detection of the distractors (e.g., Friedman et al., 2001; Berti, 2013). In addition, the novelty-P3 component, a positive deflection occurring around 300 ms after stimulus onset and highest in the frontal lobes, was found to index the involuntary switch of attention to the distractors (Escera et al., 1998, 2000; Friedman et al., 2001). Finally, the reorienting negativity (RON) component, a later negative deflection occurring around 500 ms after the stimulus onset, maximal at frontal site, was found to index the reorientation of attention back to the task after distraction (Schröger and Wolff, 1998; Schröger et al., 2000; Berti and Schröger, 2001; Wetzel et al., 2004).

### The Effect of Working Memory Load on the Processing of Auditory Distractors

Many studies have investigated how the processing of distractors is impacted by both perceptual load (Tellinghuisen and Nowak, 2003; Lavie, 2005; Parks et al., 2011; Lavie et al., 2014; Bonato et al., 2015) and working-memory load (WML; Lavie et al., 2004; Kim et al., 2005; SanMiguel et al., 2008). Lavie et al. (2004) proposed that while an increase in perceptual load may reduce distractor interference, an increase in WML may, on the contrary, increase distractor interference. However, various studies investigating the impact of WML on the processing of both visual targets and auditory distractors found opposite results (SanMiguel et al., 2008; Lv et al., 2010; Sörqvist et al., 2012). In SanMiguel et al.’s (2008) study, participants performed an auditory-visual distraction paradigm. While performing the visual task, participants had to ignore task-irrelevant auditory stimuli (i.e., 20% novel environmental sounds and 80% repetitive standard tones). The WML was also manipulated by an n-back task (Kirchner, 1958). In the low load condition (i.e., 0-back), participants had to decide whether the two digits appearing on screen at the same time were the same or different, while in the high load condition (i.e., 1-back) they had to compare the left digit appearing on the screen with the left digit seen in the previous trial. An increase of response times and a decrease of hit rate showed that participants were distracted by novel sounds. Moreover, behavioral data and an attenuation of the amplitude of the novelty-P3 showed that high WML decreased the distraction effect. In another study, Lv et al. (2010) asked participants to remember the order of three (low load) or seven digits (high load). As in SanMiguel et al. (2008), task-irrelevant auditory stimuli were played during the working memory (WM) task with 80% repetitive standard sounds and 20% novel environmental sounds. Participants responded faster and performed significantly better on the task in the low load condition than in the high load condition. Moreover, lower novelty-P3 amplitudes were found in the high WML condition in comparison to the low WML condition, leading the authors to conclude that high WML decreases the distraction effect. Finally, Sörqvist et al. (2012) measured the auditory brainstem responses (ABR; i.e., a neural signal transmitted by the cochlea to the auditory cortex via the brainstem) of participants completing a visual-verbal version of the n-back task (i.e., low load for 1-back, medium load for 2-back and high load for 3-back). They were presented with a visual sequence of letters and were asked to press the space bar on the computer keyboard when the letter was the same as the letter previously presented n letters back in the sequence. The results of this study demonstrate that a medium increase in WML (i.e., 2-back condition) may disrupt the processing of the distractor (i.e., lower ABR responses) without affecting task performance, while a significant increase of WML (i.e., 3-back condition) may not only result in lower ABR response but also in lower accuracy.

The behavioral and electrophysiological (i.e., ERPs, ABR) results of these three studies tend to confirm that high WML reduces the distraction effect. The impact of WML on distractors processing may also depend on how WM content (e.g., tones, digits, letters, words, geometric forms, etc.) overlaps task-relevant information. Stroop interference was found to increase when target types overlapped WM content (Kim et al., 2005). This result provides a suitable explanation for the discrepancy in results of studies investigating the impact of WML on auditory distractor processing (i.e., an increase of distraction vs. a decrease of distraction under high WML). However, significantly, all these experiments only used tones as auditory distractors. Linguistic distractors were studied previously by Mayer and Kosson (2004), but as far as we know the impact of WML on the processing of visual task-relevant and auditory task-irrelevant linguistic stimuli has never been investigated.

### Visual-Auditory Interference and Load on Working Memory in the Cockpit

Operating an aircraft generates a high WML, pilots have to simultaneously select, process, memorize and retrieve an important amount of information, which requires high multitasking and WM capacity (Konig et al., 2005). Previous studies in flight simulators have demonstrated the critical impact of human WM limitations on piloting performance (Taylor et al., 2000; Causse et al., 2011) and how it is likely to compromise flight safety (Borghini et al., 2014). For instance, high WML was found to affect the ability of the pilots to process ATC verbal instructions (Taylor et al., 2005) and simulated auditory alerts (Dehais et al., 2013; Giraudet et al., 2015). However, pilots find themselves confronted with false or irrelevant information (Belcastro et al., 2016). In order to maintain optimal performance, they sometimes need to insulate themselves from auditory distractors to focus on relevant visual information. For example, they have to ignore irrelevant ATC communications and unjustified warnings (e.g., ground proximity system alarm, Loomis and Porter, 1982) while always maintaining the ability to shift attention to concurrent stimuli to decide whether or not they are task-relevant. However, as previously stated, this ability to detect and process concurrent stimuli might be altered, a situation sometimes referred to as cognitive tunneling (Wickens et al., 2015).

### Present Study and Hypothesis

The present study aimed at investigating the impact of high WML on the processing of both linguistic visual targets and auditory distractors. Participants performed a simplified piloting task in which they had to control an aircraft using a joystick. They were instructed to follow one of three different colored aircraft displayed on the left of the computer screen, according to written instructions (i.e., the color corresponding to one of the aircraft) displayed in the center of the screen. Similarly to Donohue et al. (2013), each time a color was displayed on the screen, a concurrent spoken distractor (i.e., a color to be ignored) was simultaneously presented either congruently or incongruently with the written instructions. WML was manipulated via an n-back-like sub-task (i.e., the delay between the displayed instruction and its execution). In the low WML condition, participants were instructed to immediately apply the written instruction. In the high WML condition, they had to apply the 2-back written instruction. We measured piloting performance (i.e., accuracy in following the correct aircraft), ERPs, and pupil diameter.

We expected that accuracy may be higher in the congruent vs. incongruent condition. Also, the piloting task should be cognitively more demanding when the WML is high (i.e., 2-back condition) compared to when it is low (i.e., 0-back condition), thus we expected to observe higher accuracy in the low WML condition compared to the high WML condition (in line with Sörqvist et al., 2012). In addition, given that less attentional resources should be available for processing spoken distractors in the high WML condition, in line with various authors (e.g., SanMiguel et al., 2008; Lv et al., 2010; Sörqvist et al., 2012), incongruence may affect accuracy only in the low WML condition.

As pupil diameter was found to vary according to attentional effort (Smallwood et al., 2011), and task engagement (Gilzenrat et al., 2010), we predicted greater pupil diameter in response to incongruent trials compared to congruent trials (Siegle et al., 2004). This greater pupil diameter may be observed in the low WML condition only. Pupil diameter measurements have been also found to be a reliable psycho-physiological marker of WML (van Gerven et al., 2004; Lisi et al., 2015; Peysakhovich et al., 2015), with larger tonic pupil responses indicating an increase in WML. In line with previous studies, we expected to observe greater pupil diameter in the 2-back condition than in the 0-back condition.

At an electrophysiological level, we expected to observe amplitude modulations of ERP components associated with attention allocation (i.e., novelty-P3). Various studies support the fact that the P3a component and the novelty-P3 component are variations of the same potential (Spencer and Polich, 1999; Simons et al., 2001; Polich and Comerchero, 2003; Polich, 2007). Since no novel auditory distractors were used in the present task, we chose to use the generic term P3a and not novelty-P3 when referring to this component. Based again on previous results showing that an increase in WML may lower the distraction effect (SanMiguel et al., 2008; Lv et al., 2010; Sörqvist et al., 2012), we predicted greater P3a amplitudes in response to incongruent trials than to congruent trials in the low WML condition only, reflecting an involuntary switch of attention to the spoken distractors (e.g., Escera et al., 1998, 2000; Friedman et al., 2001). In addition, spoken distractors seem to lead to involuntary semantic evaluation (Parmentier, 2008; Parmentier et al., 2011; Parmentier and Hebrero, 2013). In order words, spoken distractors are, at least partly, processed. Based on these previous results, we predicted amplitude modulations of ERPs associated with language processing (i.e., N400 component). The N400 component is a negative deflection reaching the scalp around 400 ms after stimulus onset, is highest at central-parietal sites (for a review see Kutas and Federmeier, 2011), and was found to index sematic incongruence processing (Pickering and Schweinberger, 2003). Consequently, we predicted greater N400 amplitudes in response to incongruent trials compared to congruent trials (Kutas and Hillyard, 1980; Kutas and Federmeier, 2011), indicating the processing of both written instructions and spoken distractors (in line with Donohue et al., 2012). As for the P3a, we may also observe N400 amplitude differences in response to incongruent trials vs. congruent trials in the low WML condition only. Finally, we predicted a general effect of WML with lower P3a/N400 amplitudes in the 2-back condition than in the 0-back condition (SanMiguel et al., 2008; Lv et al., 2010).

## Materials and Methods

### Participants

Participants were 24 healthy volunteers (mean age = 24.6, *SD* ± 1.86), all native French speakers. They were recruited at the Institut Supérieur de l’Aéronautique et de l’Espace (ISAE) and were familiar with the aeronautical domain. All were right-handed as assessed by the Edinburgh Handedness Inventory (Oldfield, 1971), had normal auditory acuity and normal or corrected-to-normal vision. None of the participants reported a prior history of neurological disorder. All participants were informed of their rights and gave written informed consent for participation in the study according to the Helsinki Declaration. The research was carried out fulfilling ethical requirements in accordance with the standard procedures of the University of Toulouse. The experimental protocol was reviewed and approved by a national ethic committee (CEEI/IRB00003888).

### Material

The piloting task was displayed on a 22” monitor (1680 × 1250) located at a distance of approximately 70 cm from the participants. The screen luminance and the piloting task were identical in all experimental conditions. As a consequence, no confounding effect of light could have jeopardized pupil measurements. Spoken names of colors (i.e., gray, red, blue, yellow, green) were presented via two stereo speakers, positioned on each side of the computer monitor. They were recorded using a synthetic voice taken from the French Voxygen startup website.^1^ Throughout the entire experiment, both pupil diameter variations and EEG signals were recorded (see “Electroencephalography” and “Pupillometry” Sections).

### Experiment Design

We used a full factorial design with two within-participant factors: WML (Load: Low, High) and the congruency of task-irrelevant auditory distractor (Congruency: Congruent, Incongruent). Participants performed two blocks of 250 trials each, which only presented either “Low” or “High” load trials. Block order was counter-balanced among participants. The congruency of the task-irrelevant auditory distractor was randomly determined per trial. We computed accuracy for each condition as a percentage of correctly targeted aircraft. We considered that an aircraft was correctly followed if the vertical distance between the user’s aircraft and the target one was less than 100 pixels for at least 90% of the trial length.

### Task and Stimuli

The task involved controlling an aircraft with a joystick in order to follow one of three possible aircraft that were defined by unique colors. The color name corresponding to the color of the aircraft to target was presented in black ink every 4500 ms in the center of the screen for 1000 ms (i.e., written instructions). In addition, a task-irrelevant auditory distractor (i.e., spoken distractor) was also presented simultaneously for 280 ms (i.e., visual-auditory Stroop paradigm; Roelofs, 2005). These written instructions and spoken distractors created four different trial combinations. In the first combination occurring 10% of the time, the spoken and the written color names (i.e., blue, red, green or yellow) were the same (i.e., congruent trials). In the second combination also occurring 10% of the time, the spoken and the written color names differed from one another (i.e., incongruent trials). In the third combination occurring 10% of the time, the spoken color name did not correspond to any aircraft color (i.e., neutral trials). And finally, in the fourth combination occurring 70% of the time, the spoken color name was “gray” (i.e., standard trials). The neutral and standard trials were not analyzed. These two trial combinations were used to inhibit habituation effects and create a rarity effect toward the congruent/incongruent distractors, respectively.

**Figure 1:**
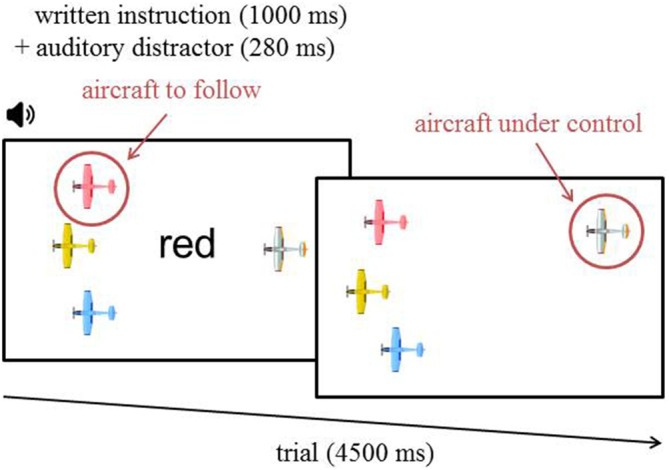
**Time course of a task trial.** The written instruction was displayed for 1000 ms while the auditory distractor was played for 280 ms. In this particular example, the written target color is “red”. Consequently, a congruent auditory distractor would be “red”. “Yellow” or “blue” would be incongruent distractors, and “green” would be neutral. The standard distractor was “gray” throughout the whole experiment.

The three aircraft to target were displayed on the left of the computer screen. Figure 1 shows the layout of the presentation display. The colors of the three aircraft were randomly chosen for each block among four possible colors: red, blue, yellow and green. The initial horizontal position of the targeted aircraft and the control aircraft were equidistant from the center and the edges of the display. They were respectively positioned 30% from the left/right borders of the screen. In order to create a continuous, engaging, and dynamic interaction with the task, every 50 ms the position of the three aircraft on the left changed, a random shift up to 12 pixels vertically and up to 2 pixels horizontally was applied. The amplitude of this shift was chosen by a moving average filter (of 10th order) of randomly generated numbers (from −1 to 1 to choose the proportion of the greatest authorized shift). A small jitter was also added to the aircraft under control, with a maximum authorized shift up to five pixels vertically and up to one pixel horizontally, so it would be unstable and require continuous control.

**Figure 2:**
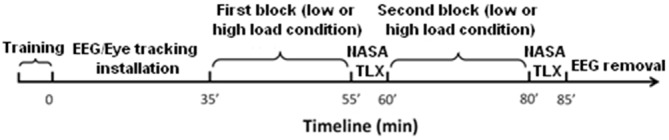
**Timeline of the experiment.** The whole procedure lasted 85 min, including 5 min training, 30 min to install the EEG sensors, 40 min of EEG recording and 10 min to fill the two NASA TLX questionnaires.

Task difficulty was manipulated in terms of WML and the tracking task was designed to be similar to an n-back task. Two difficulty levels corresponded to the delay between the displayed instruction and its execution. Contrary to the classic n-back paradigm in which a participant has to indicate if the current stimulus matches one from n steps earlier in the sequence, our participants had to target the aircraft corresponding to the current written instruction (*n* = 0) in the low WML condition or corresponding to the instruction presented two trials before (*n* = 2) in the high WML condition. After each block, participants filled out the NASA Task Load Index questionnaire (NASA TLX; Hart and Staveland, 1988), see Figure 2. This questionnaire provides an evaluation of the subjective mental demand elicited by the task for each level of difficulty.

We did not manipulate piloting complexity *per se*, the control of the aircraft remained constant. Neuroergonomically, this task was designed to recreate an ecological context with an engaging, dynamic and complex situation. Similar to piloting, the participants had to continuously control the trajectory of their aircraft and had to remain responsive to the written and verbal instructions under various WML conditions. In addition, the task also reproduced the multiple conflicting warnings that can confuse crews (Belcastro et al., 2016). Given the voluntary complexity of the task, we did not intend to specifically separate all cognitive processes at each time point. However, the main cognitive abilities engaged during the task were visuospatial (monitoring the position of the aircraft), psychomotor (control of the aircraft) and attentional (toward the instructions).

### Procedure

Participants were comfortably seated in an armchair in a sound-dampened experimental room. The room had no windows and the light was kept constant and moderate. After the training session, they were equipped with the EEG electrode cap as well as the electrooculographic electrodes for blink and saccade detection. Eye tracker calibration was performed to record participants’ pupil diameter. Participants then performed the four experimental blocks. The instructions were generated so the participant had to change target aircraft every trial. The tracking was continuous during the whole task; when a new instruction was displayed, participants still followed the previous aircraft until they processed the new instruction and switched to the corresponding aircraft. Participants could not repeat aloud the instructions and they were instructed to avoid moving and talking. After each of the four blocks, participants filled out the NASA-TLX.

### Electroencephalography

EEG was amplified and recorded with an ActiveTwo BioSemi system (BioSemi, Amsterdam, Netherlands) from 30 Ag/AgCl active electrodes mounted on a cap and placed on the scalp according to the International 10–20 System (FP1, FP2, AF3, AF4, F7, F3, Fz, F4, F8, FC5, FC1, FC2, FC6, CP5, CP1, Cz, CP2, CP6, P7, P3, Pz, P4, P8, T7, T8, PO3, PO4, O1, Oz, O2) plus two sites below the eyes for monitoring eye movements. Analyses were focused on 23 electrodes of interest. Two additional electrodes placed close to Cz, the Common Mode Sense (CMS) active electrode and the Driven Right Leg (DRL) passive electrode, were used to form a feedback loop that maintains the average potential of the participant as close as possible to the AD-box reference potential. Electrode impedance was kept below 5 kΩ for scalp electrodes, and below 10 kΩ for the four eye channels. Skin-electrode contact, obtained using electro-conductive gel, was monitored, keeping voltage offset from the CMS below 25 mV for each measurement site. All the signals were (DC) amplified and digitized continuously with a sampling rate of 512 Hz with an anti-aliasing filter with 3 dB point at 104 Hz (fifth-order sinc filter); no high-pass filtering was applied online. The triggering signals to each word onset were recorded on additional digital channels. EEG data was off-line re-referenced to the average activity of the two mastoids and band-pass filtered (0.1–40 Hz, 12 dB/octave), given that for some participants the low-pass filter was not effective in completely removing the 75 Hz artifact. Epochs were time-locked to instructions onset and extracted in the interval from −200 ms to 800 ms. The 200 ms pre-stimulus baseline was used in all analyses. Given their synchronicity, we could not dissociate the respective contributions of written instructions and auditory distractors on the ERPs. Segments with excessive blinks and/or artifacts (such as excessive muscle activity) were eliminated off-line before data averaging. The lost data (due to artifacts) represented 7%.

### Pupillometry

The diameter of participants’ left pupil was continuously recorded with a remote SMI RED eye-tracker (SensoMotoric Instruments GmbH, Germany) at a sampling rate of 500 Hz. Before each condition, participants performed a 5-point calibration procedure. The continuous pupillary recordings were cleaned for blink artifacts using linear interpolation, including adjacent 40 ms from each side to avoid eyelid closure artifacts. The data was then filtered with a “two pass” 9-point filter (low-pass) and segregated into trials by conditions. A trial was validated for the statistical analysis if the time spent blinking during the trial did not exceed 50% (i.e., 2250 ms). This resulted on average in 87% (*SD =* 18%) of validated trials per condition and was not dependent on the condition.

### Statistical Analyses

Statistical analyses were performed using Statistica 10 (StatSoft). Differences between the experimental conditions were investigated by using analysis of variance (ANOVA) followed by Tukey’s honestly significant difference (HSD) *post hoc* testing.

## Results

### Performance

**Figure 3:**
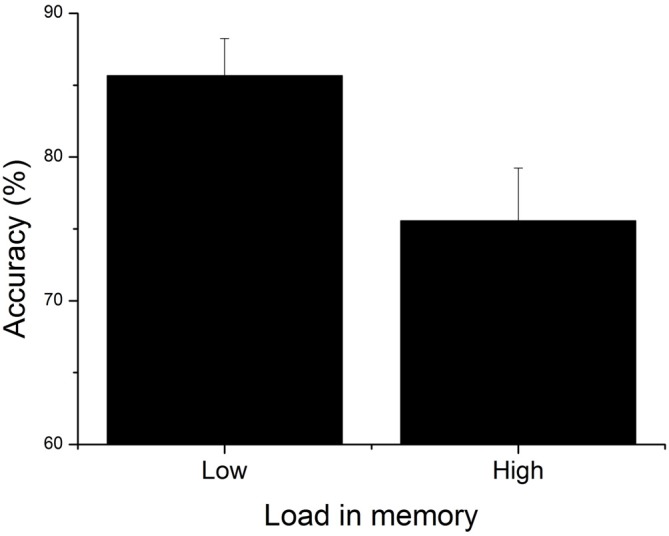
**Mean accuracy for low and high load conditions.** Error bars indicate SEM.

**Figure 4:**
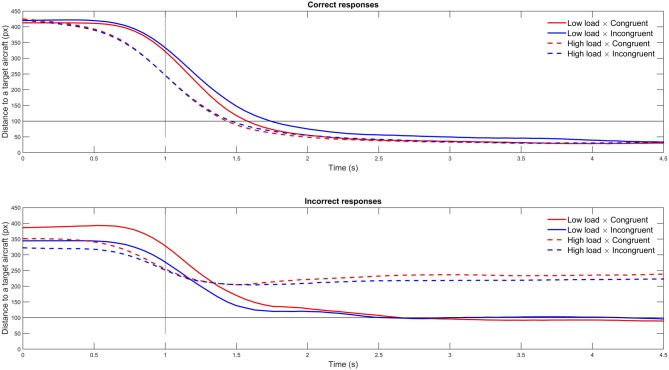
**Mean distance to a target aircraft per condition for correct and incorrect trials.** The black horizontal line depicts the threshold below which the aircraft was considered as selected. The vertical dashed black line depicts the time when the written instruction disappeared from the computer screen.

A 2 × 2 (Congruency [Congruent; Incongruent] × Load [Low; High]) repeated measures ANOVA revealed a main effect of WML on piloting performance (*F*_(1,23)_ = 7.79, *p* < 0.05, $\eta_{p}^{2}$ = 0.25); the participants were better at aircraft targeting in the low WML condition (*M* = 85.65, *SD* ± 13.26) compared to the high WML condition (*M* = 75.55, *SD* ± 16.65; see Figure 3). On the contrary, we found no effect of congruency (*F*_(1,23)_ = 0.08, *p* = 0.78, neither Load × Congruency interaction (*F*_(1,23)_ = 1.18, *p* = 0.29, $\eta_{p}^{2}$ = 0.05) on piloting performance. Figure 4 shows the temporal evolution of the distance between the user’s and the target aircraft for each condition and correct and incorrect trials. This fine-grained analysis confirms that poorer piloting performance in the high WML condition cannot be associated with slow or inaccurate following of the correct aircraft. The decline of piloting performance under high WML was due to incorrect targeting (i.e., the inability to correctly encode and retrieve the aircraft to target). For the incorrect trials, the distance between the target aircraft and the user’s was indeed kept constant at about 220 pixels corresponding to a wrong aircraft. On the contrary, the rare incorrect trials under low WML are mainly due to poor following of the correct aircraft (the average distance oscillates just above the 100 pixel threshold).

### NASA-TLX Questionnaire

**Figure 5:**
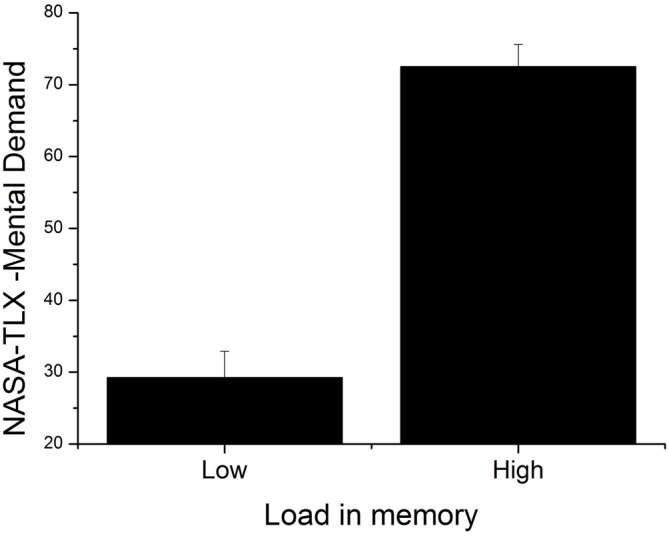
**Mean index of perceived mental demand for low and high load conditions.** Error bars indicate SEM.

The 2 × 6 (Load [low, high] × NASA-TLX dimensions [Mental Demand, Physical Demand, Temporal Demand, Performance, Effort, Frustration]) repeated measures ANOVA showed a significant effect of load (*F*_(1,23)_ = 32.68, *p* < 0.001, $\eta_{p}^{2}$ = 0.59). Participants evaluated the high WML condition as more mentally demanding, see Figure 5. The ANOVA also showed a significant effect of the NASA-TLX dimension (*F*_(1,23)_ = 8.23, *p* < 0.001, $\eta_{p}^{2}$ = 0.26). The three dimensions with the highest scores were (in descending order): Performance, Effort and Mental Demand. Finally, a Load × NASA-TLX dimensions interaction was found (*F*_(5,15)_ = 7.83, *p* < 0.001, $\eta_{p}^{2}$ = 0.25). Most notably, in the high WML condition, the Mental Demand dimension was rated higher on average than all other conditions (*M* = 72.50, *SD* = 15.28). However, this is statistically significant only when compared to Physical Demand and Temporal Demand (HSD: *p* < 0.01 in both comparisons).

### Electroencephalography

**Figure 6:**
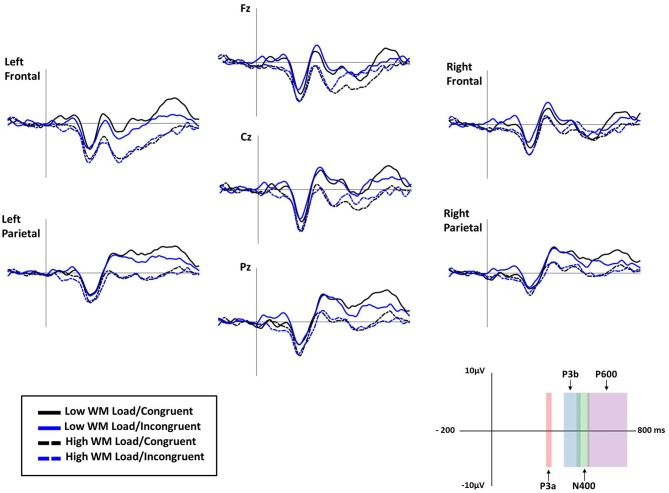
**Grand average ERP waveforms for low working memory (WM) load/congruent (black line), low WM load/incongruent (blue line), high WM load/congruent (dashed black line) and high WM load/incongruent trials (dashed blue line) at Fz, Cz, Pz, and left frontal, left parietal, right frontal and right parietal electrode-clusters.** In the bottom right corner, the *x*-axis displays the 4 ERP components (vertical bar indicates the onset of both the written instruction and the auditory distractor) and the *y*-axis displays amplitude in microvolts. Negative is plotted down.

Because of EEG recording issues, data were not available for one participant (corrupted data). First, five 3 × 2 × 2 (Electrode [Fz, Cz, Pz] × Congruency [congruent, incongruent] × Load [low, high]) repeated measure ANOVAs were conducted to assess mean amplitudes of MMN, P3a, P3b, N400 and P600 components on the three midline electrodes. ERPs time windows were determined through both literature and visual analysis of the peak amplitudes. Second, in order to investigate possible topographical differences for these ERPs, the remaining electrodes were collapsed into four regions of interest of five electrodes each (see Siyanova-Chanturia et al., 2012): Left Anterior (AF3, F7, F3, FC1, FC5), Right Anterior (AF4, F4, F8, FC2, FC6), Left Posterior (CP2, CP6, P4, P8, PO4) and Right Posterior (CP1, CP5, P3, P7, PO3). A 4 × 2 × 2 (Region [Left Anterior, Right Anterior, Left Posterior, Right Posterior] × Congruency [congruent, incongruent] × Load [low, high]) ANOVA was conducted. See Figure 6 for grand average ERP waveforms.

#### MMN (200–240 ms Time Window)

The MMN amplitude was assessed in terms of the mean amplitude in the 200–240 ms time window. The statistical analysis revealed no significant main effect or interaction (*p*s > 0.05).

#### P3a (300–330 ms Time Window)

The P3a amplitude was assessed in terms of mean amplitude in the 300–330 ms time window. The analysis revealed a main effect of load (*F*_(1,22)_ = 8.13, *p* < 0.01, $\eta_{p}^{2}$ = 0.27), with a greater positivity in the low WML (*M* = 3.34 μV, *SD* ± 6.77) than in the high WML condition (*M* = 0.10 μV, *SD* ± 5.92). The analysis also revealed a significant Electrode × Congruency interaction (*F*_(2,44)_ = 3.23, *p* < 0.05, $\eta_{p}^{2}$ = 0.13), with a greater positivity for incongruent trials (*M* = 1.21 μV, *SD* ± 6.64) than for congruent trials (*M* = 0.39 μV, *SD* ± 7.11), (HSD: *p* < 0.05) at Fz. No significant differences were found at Cz (HSD: *p* = 0.84) and at Pz (HSD: *p* = 0.13).

#### P3b (400–490 ms Time Window)

The P3b amplitude was assessed in terms of the mean amplitude in the 400–490 ms time window. The analysis revealed a main effect of load (*F*_(1,22)_ = 5.03, *p* < 0.05, $\eta_{p}^{2}$ = 0.19), with a greater positivity in the low WML condition (*M* = 1.27 μV, *SD* ± 7.89) than in the high WML condition (*M* = −2.03 μV, *SD* ± 5.24). The analysis also revealed an Electrode × Congruency interaction (*F*_(2,44)_ = 3.52, *p* < 0.05, $\eta_{p}^{2}$ = 0.14), with a greater positivity in response to incongruent trials (*M* = −2.08 μV, *SD* ± 6.28) compared to congruent trials (*M* = −3.22 μV, *SD* ± 7.76; HSD: *p* = 0.01) at Fz. No significant differences were found at Cz (HSD: *p* = 0.21) and at Pz (HSD: *p* = 0.54).

#### N400 (470–540 ms Time Window)

The N400 was assessed in terms of the mean amplitude in the 470–540 ms time window. The analysis revealed a significant Electrode × Load interaction (*F*_(2,44)_ = 9.45, *p* < 0.001, $\eta_{p}^{2}$ = 0.30), with greater negativities in the high WML condition (Fz: *M* = −3.70 μV, *SD* ± 6.00; Cz: *M* = −2.91 μV, *SD* ± 4.93; Pz: *M* = −1.05 μV, *SD* ± 4.26) than in the low WML condition (Fz: *M* = −2.09 μV, *SD* ± 7.72; Cz: *M* = −0.36 μV, *SD* ± 7.52; Pz: *M* = 2.80 μV, *SD* ± 7.67; HSD: *p*s < 0.001) on Fz, Cz and Pz. The analysis also revealed a significant Electrode × Congruency interaction (*F*_(2,44)_ = 8.95, *p* < 0.001, $\eta_{p}^{2}$ = 0.29), with a greater N400 amplitude for incongruent trials (*M* = 0.48 μV, *SD* ± 6.12) than for congruent trials (*M* = 1.27 μV, *SD* ± 6.82) at Pz (HSD: *p* < 0.05). The opposite pattern was found at Fz (incongruent trials: *M* = −2.17 μV, *SD* ± 6.48; congruent trials: *M* = −3.62 μV, *SD* ± 7.34; HSD: *p* < 0.001; this apparent contradiction for decreased N400 for incongruent trials in Fz can be explained by the previous more positive P3a on this electrode in this condition). No difference was found at Cz (incongruent trials: *M* = −1.33 μV, *SD* ± 6.16; congruent trials: *M* = −1.94 μV, *SD* ± 6.78, (HSD: *p* = 0.11). The topographical analysis revealed no significant effect or interaction.

#### P600 (530–750 ms Time Window)

The P600 was assessed in terms of the mean amplitude in the 530–750 ms time window. The analysis revealed a main effect of load (*F*_(1,22)_ = 8.15, *p* < 0.01, $\eta_{p}^{2}$ = 0.27), with a greater positivity in the low WML condition (*M* = 2.44 μV, *SD* ± 7.08) than in the high WML condition (*M* = −1.02 μV, *SD* ± 5.18). A significant Electrode × Congruency interaction (*F*_(2,44)_ = 5.53, *p* < 0.01, $\eta_{p}^{2}$ = 0.20), was found with a greater positivity observed for congruent trials (*M* = 2.97 μV, *SD* ± 6.45) compared to incongruent trials (*M* = 1.62 μV, *SD* ± 5.43) at Pz (HSD: *p* < 0.001), but not at Fz (HSD: *p* = 0.44) nor at Cz (HSD: *p* = 0.75). The topographical analysis revealed a significant Load × Region interaction (*F*_(3,66)_ = 8.83, *p* < 0.001, $\eta_{p}^{2}$ = 0.29), with greater positivity in both left and right posterior regions in the low load condition (respectively: *M* = −0.28 μV, *SD* ± 3.77; *M* = −0.14 μV, *SD* ± 3.82) than in the high WML condition (respectively: *M* = −1.14 μV, *SD* ± 7.72, *p* < 0.001; *M* = −1.61 μV, *SD* ± 8.34; HSD: *p* < 0.001). The analysis also revealed a second significant Load × Congruency interaction (*F*_(1,22)_ = 5.11, *p* < 0.05, $\eta_{p}^{2}$ = 0.19), with a greater positivity in response to congruent trials compared to incongruent trials in the low WML condition (respectively: *M* = −0.45 μV, *SD* ± 6.43; *M* = −1.79 μV, *SD* ± 8.47; HSD: *p* < 0.05), but not in the high WML condition (respectively: *M* = −0.67 μV, *SD* ± 3.89; *M* = −0.24 μV, *SD* ± 4.09; HSD: *p* = 0.44). Moreover, a greater positivity was also found for incongruent trials in the high WML condition (*M* = −0.24 μV, *SD* ± 4.09) than in the low WML condition (*M* = −1.79 μV, *SD* ± 8.47; HSD: *p* < 0.05).

### Pupillometry

Two 2 × 2 (Load [low, high]) × Congruency [congruent, incongruent]) repeated measure ANOVAs were carried out on the mean value of 1.5-s recording starting from 1-s post-stimulus for tonic pupil response (absolute diameter) and phasic pupil response (relative dilation). This interval largely includes the peak of pupillary reaction known to appear about 1200–1500 ms post-stimulus (Beatty and Lucero-Wagoner, 2000). We used the mean value of 500 ms pre-stimulus as a baseline value for statistical analyses of the phasic pupil response.

#### Tonic Pupil Response

**Figure 7:**
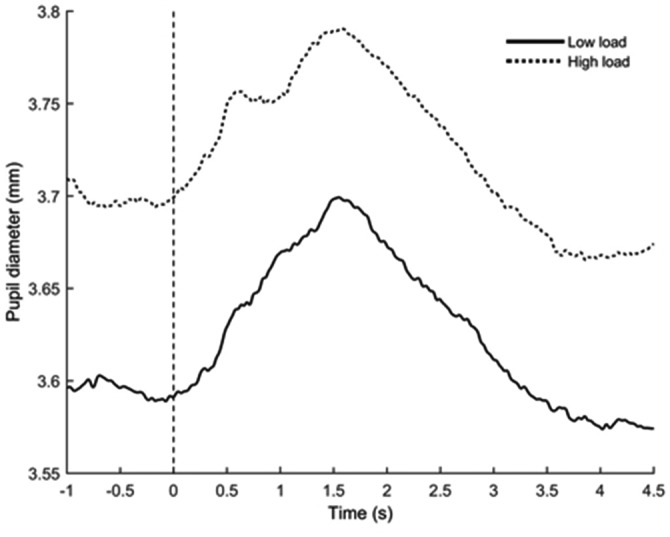
**Grand-averaged mean pupil diameter during trials for low load (solid black line) and high load (dotted black line) conditions.** The horizontal axis denotes time in seconds (s) and the vertical axis denotes pupil diameter in millimeters (mm). Zero on the timeline indicates the onset of both the written instruction and the auditory distractor.

The ANOVA showed a significant effect of WML (*F*_(1,23)_ = 9.14, *p* < 0.01, $\eta_{p}^{2}$ = 0.28; see Figure 7). The high WML condition elicited larger tonic pupil response (*M* = 3.76 mm, *SD* ± 0.11) compared to the low WML condition (*M* = 3.68 mm, *SD* ± 0.11). No Congruency (*F*_(1,23)_ = 1.82, *p* = 0.19, $\eta_{p}^{2}$ = 0.07), nor Load × Congruency interaction (*F*_(1,23)_ = 0.85, *p* = 0.37, $\eta_{p}^{2}$ = 0.04] was found.

#### Phasic Pupil Response

No significant effects of WML (*F*_(1,23)_ = 0.85, *p* = 0.37, $\eta_{p}^{2}$ = 0.04), congruency (*F*_(1,23)_ = 0.53, *p* = 0.47, $\eta_{p}^{2}$ = 0.02), or Load × Congruency interaction (*F*_(1,23)_ = 0.24, *p* = 0.63, $\eta_{p}^{2}$ = 0.01) were found for phasic pupil response.

## Discussion

Operating an aircraft is cognitively demanding and requires high multitasking and WM capacities (Konig et al., 2005). Pilots have to simultaneously process, memorize and retrieve an important amount of visual and auditory information. In addition to this high cognitive load, pilots sometimes have to ignore irrelevant auditory distractors such as background ATC communications and false alarms. Paradoxically, they must remain responsive to unexpected stimuli at all times. Previous studies emphasized that attention-demanding settings that generate a high WML can impair the perception of unexpected/irrelevant stimuli (Berti and Schröger, 2003; Sörqvist et al., 2012). However, the impact of WML on the processing of linguistic material has rarely been tested in an explicit way. In the present study, participants completed a cross-modal version of the Stroop task paradigm (Donohue et al., 2013) adapted to a dynamic piloting task in combination with ERP and pupillary measurements. They were asked to take into account a written target instruction (i.e., the name of a color) and to ignore a concurrent spoken distractor (i.e., also a color). We investigated how WML modulated the processing of both target and distractors and to what extent it affected piloting performance (this latter being dependent on the processing/maintenance/retrieval of written instructions). Overall results revealed a subtle effect of congruency that was observable only at an electrophysiological level, an interaction between congruency and load on P600 amplitude, and a major effect of WML at behavioral, electrophysiological, and autonomic levels.

### Impact of the Congruency

At a behavioral level, the results revealed no main effect of the congruency between the written target and the spoken distractor, indicating that the latter does not interfere enough with the processing of the written instruction to affect piloting performance. This absence of interference at a behavioral level may be due to some limitations in the experimental paradigm. First, a recent study showed that distraction is maximal when an auditory distractor is presented 400 ms before the onset of the visual information of interest, but that it is significantly reduced when both stimuli of interest and the distractor are presented at the same time (Donohue et al., 2013). Since in the present study, ERP measurements were performed, it would have been complex to interpret electrophysiological results if the distractor and the target had been presented at different times. Presenting the spoken distractor before the written instruction could have led to a greater distraction effect, observable at the behavioral level. Second, in general, the distraction effect is observable on reaction times rather than accuracy measures. Longer reaction times reflect the penalty yielded by the involuntary orientation of attention to and away from deviant sounds (Parmentier, 2008). Given the continuous control of the aircraft trajectory with the joystick, it was not possible to measure reaction times accurately. We could have captured variations of reaction times if the task allowed such measurements.

However, electrophysiological data demonstrate the increased complexity for processing the written instructions when incongruent spoken distractors were presented simultaneously, with greater P3a and N400 amplitudes in incongruent trials compared to congruent trials. According to Polich (2007), the P3a component may be generated when focal attention on the task-relevant stimuli is captured by a distractor; and indexes the automatic allocation of attentional resources to the distractor at the expense of goal-relevant stimuli. Greater P3a amplitudes in response to incongruent trials may reflect the recruitment of supplementary attentional resources and the involuntary orienting of attention to the spoken distractors (Escera et al., 1998, 2000; Friedman et al., 2001).

The N400 component was found to index semantic incongruence processing (Kutas and Federmeier, 2011). In line with the literature (Hanslmayr et al., 2008; Kutas and Federmeier, 2011; Donohue et al., 2012), greater N400 amplitudes were found at parietal sites in incongruent trials compared to congruent trials. Some behavioral studies have shown that not only are attentional resources allocated to spoken distractors, but that they also lead to an involuntary semantic evaluation (Parmentier, 2008; Parmentier et al., 2011; Parmentier and Hebrero, 2013). The N400 component may index this involuntary semantic analysis of the spoken distractor. However, as the processing of the spoken distractor was not sufficient to trigger significant distraction observable at a behavioral level, we conclude that the mobilization of additional attentional resources indexed by the P3a component was sufficient to process both the relevant written instruction and the irrelevant spoken distractor.

### Impact of the Working-Memory Load

At both behavioral and subjective levels, a higher level of WML was found to decrease the accuracy and was associated with a higher perceived mental demand as shown by the NASA-TLX questionnaire. Moreover, the pupil diameter was also modulated by WML, with greater tonic pupillary responses under high WML compared to low WML, thus objectively confirming the increased task difficulty in the high WML condition.

Given that difficulty levels were generated using an n-back-like task, the tonic pupil diameter indicates the WML that was maintained throughout the block. Thus, when segregated into trials, the tonic reaction is always higher during the high WM condition. Pupil diameter was found to be correlated with WML and task difficulty (Kahneman and Beatty, 1966; Beatty and Lucero-Wagoner, 2000; Karatekin et al., 2007; Causse et al., 2010; Peysakhovich et al., 2015). Greater pupil diameter observed during trials of high WML provides additional evidence that higher WML increases the attentional resources allocated to the task. The present study also demonstrates that it is possible to measure pupil dilation relative to variations in WML in a task that requires natural eye movement if luminosity remains constant.

At an electrophysiological level, an increase in WML was found to affect the allocation of attentional resources (i.e., P3a and P3b components) and the semantic processing of both the written instruction and the spoken distractors (i.e., P600 component). The amplitudes of both the P3a and P3b components were lower in the high load condition than in the low load condition. An increased WM demand in the n-back task was previously shown to enlist attentional resources and processing capacity away from the matching subtask (i.e., the comparison process; Watter et al., 2001) and was associated to reduced P300 amplitudes. For this reason, decreased amplitudes of P3a and P3b in the high WML condition of the present study is interpreted as an “overall” alteration of the ability to orient attention and process environmental stimuli, including the critical written instruction. A classic hypothesis postulates that the P3a component originates from stimulus-driven frontal attention mechanisms when sufficient attentional focus is engaged (Polich, 2007). Recent research has shown that the P3a component indicates information selection within the WM (Berti, 2016). The P3b component originates from temporal-parietal activity when subsequent attentional resources promote context updating operations and memory processing (Knight, 1996; Brázdil et al., 2001; Polich, 2007). The P3b component is also considered to indicate stimulus analysis and response initiation (Verleger et al., 2005). Lower P3a and P3b responses in the 2-back condition indicate the mobilization of resources that cannot be allocated to processing the current written instruction since these resources are utilized by complex operations in WM. An increased N400 negativity was found at parietal sites in the high load condition compared to the low load condition. However, this increased negativity could be attributable to a decrease in amplitude of both P3a and P3b components preceding the N400 component. Therefore, it is difficult to draw definitive conclusions on the impact of load on this component.

Our results also revealed an unpredicted decrease in amplitude of the P600 in high WML compared to the low WML condition. The P600 component is a positive deflection occurring around 500 ms after stimulus onset and is known to reflect language revision processes (Friederici, 1995; Kaan et al., 2000; Papageorgiou et al., 2001). Interestingly, a recent study suggests that the P600 component may also index attention reorientation processes (Sassenhagen and Bornkessel-Schlesewsky, 2015). While the voluntary reorientation of attention was found to be indexed by the RON component (e.g., Schröger and Wolff, 1998; Schröger et al., 2000; Berti and Schröger, 2001; Wetzel et al., 2004), occurring around 400 ms after stimulus onset for simple stimuli (i.e., tones), in the present study, this process step appears to be delayed for linguistic stimuli (i.e., words) and indexed by the P600 component. It is likely that both the voluntary reorientation of attention back to the written instruction and its reanalysis/rechecking were affected in the high WML condition because fewer WM resources were available. This interpretation corresponds with a recent study showing that patients with WM deficits demonstrate lower P600 amplitudes (El-Kholy et al., 2012).

### Interaction between Load and Congruency

At a behavioral level, the results of the present study revealed no interaction effect between the WML and target/distractor congruency. Previously mentioned limitations in the experimental paradigm could have contributed to this null effect. At an electrophysiological level, congruency was found to modulate the amplitude of the P600 component only in the low WML condition, with lower P600 responses in incongruent trials compared to congruent trials. Moreover, lower P600 amplitudes were found in response to incongruent trials in the low WML condition compared to the high WML condition. Taken together, these results are consistent with the idea that the interference effect is reduced as WML increases (e.g., SanMiguel et al., 2008; Lv et al., 2010; Sörqvist et al., 2012; Scharinger et al., 2015). We assume that attentional resources in WM were not sufficient to intensively process auditory distractors in the high WML condition. Consequently, no congruency effect was observed and the voluntary reorientation of attention back to the target instruction was easier. Higher task engagement generated by high WML condition tended to further reduce the effect of distraction observable only at an electrophysiological level. According to Kim et al. (2005), WML can either impair or benefit attentional selection depending on whether it overlaps with target/distractor processing or not. Their results showed that Stroop interference increased when the type of WML overlapped with the type of information required for the task. At the same time, Stroop interference decreased when the type of WML overlapped with distractor processing. In the present study, WML was elicited by exactly the same type of content as the targets/distractors (i.e., verbal stimuli). As a consequence, WML most likely impacted the processing of both written instructions and distractors, as shown by the decline in piloting performance and the mitigation of interference caused by the incongruent distractor, demonstrated by the P600 results.

We found results in line with previous studies (SanMiguel et al., 2008; Lv et al., 2010; Sörqvist et al., 2012) but also apparently in contradiction with the load theory, which predicts that high WML increases distractor interference by impeding inhibitory cognitive control (de Fockert et al., 2001; Lavie et al., 2004; Woodman and Luck, 2004). These contradictory results could possibly be explained by differences in the experimental paradigm. In the studies finding that WML enhances distraction, two independent tasks were combined: a “WM task” and a “selective attention task”. In these experiments, a trial consisted in: first, a memorization phase (e.g., memorizing a list of digits), second, a selective attention task (e.g., classifying a written list of famous names such as pop stars or politicians while ignoring distractor faces), and third, a memory probe (e.g., reporting the digit that followed a probe in the memory set presented at the beginning of the trial). However, in the studies in which high WML was found to reduce the distraction effect, including the present study, the WM task and the selective attention task were nested. In other words, when the distraction task and the WM task were concomitant, WML was found to lower distractor interference, while when they were not, WML enhanced distractor interference. Future studies should test this hypothesis by comparing the effect of delayed vs. concomitant WML on distractor processing. Also, in order to get closer to the real piloting situation, future works should use spoken material conveying relevant, neutral or irrelevant information to the piloting task in order to investigate top-down processing modulations associated with spoken information according to their value to the focal task.

## Conclusion

In the present study, we adapted a visual-auditory version of the Stroop paradigm (Donohue et al., 2013) to a dynamic simulated piloting task in combination with ERPs and pupillary measurements. WML was also manipulated using an n-back task. Electrophysiological results revealed that more attentional resources were mobilized during incongruent trials (i.e., P3a component) and that the incongruence between written instructions and spoken distractors was detected (i.e., N400 component), suggesting that spoken distractors were semantically processed. This result confirms previous behavioral findings showing that not only are attentional resources allocated to spoken distractors but that they also lead to an involuntary semantic evaluation of the latter (Parmentier, 2008; Parmentier et al., 2011; Parmentier and Hebrero, 2013). However, the semantic processing of distractors was not sufficient to impair task accuracy, probably thanks to the mobilization of supplementary attentional resources that enabled participants to process both the target and the incongruent distractor.

Overall, high WML disrupted the processing of both the visual target instruction and the spoken distractors. High WML provoked a decline in task accuracy and increased pupil diameter. At an electrophysiological level, an alteration of the ERPs component was found when the WML was high. In particular we found lower P3a/P3b responses indexing the mobilization of resources by the WM task that could not be allocated to orient the attention and process environmental stimuli, including the critical written instruction. We also found lower P600 responses, showing the impairment of voluntary reorientation of attention back to the processing of written instruction, thus altering the reanalysis/rechecking process. In addition, lower P600 responses in incongruent trials than to congruent trials were significant in the low WML condition only, indexing an easier voluntary reorientation of attention back to the target instruction because interference was reduced in the high WML condition. Our electrophysiological results can be related to a recent study (Scheer et al., 2016) that support a three-stage distraction model with ERPs that reflect the post-sensory detection of the task-irrelevant stimulus, engagement, and re-orientation back to the relevant task. They showed that the difficulty of a steering task not only diminished the amplitudes of early P3, late P3 but also the re-orientation negativity (RON) to the steering task (reorientation being rather indexed by P600 component in our study). Our results are also consistent with theories such as enhancing inhibitory control (Scharinger et al., 2015) and the task engagement/distraction trade-off model (Sörqvist and Rönnberg, 2014) with the idea that an higher cognitive engagement in a task can diminish the distractibility and responsiveness to additional stimuli. From an operational point of view, we confirm that high WML can compromise the ability of pilots to process, maintain, and execute ATC verbal instructions (2005) and to react to critical auditory alerts (Giraudet et al., 2015). We also demonstrate that P300 and P600 components are good candidates to detect variations in WM demand and that they allow estimation of its impact on the processing of linguistic stimuli.

## Author Contributions

MC: designed the experiment, conducted data analysis, interpreted the data and wrote the manuscript; EFF: administered the experiment, conducted data analysis, interpreted the data and wrote the manuscript; VP: designed the experiment, developed the experimental task, conducted data analysis and wrote the manuscript.

## Conflict of Interest Statement

The authors declare that the research was conducted in the absence of any commercial or financial relationships that could be construed as a potential conflict of interest.

## Footnotes

^1^https://www.voxygen.fr/fr
